# rSodC is a potential antigen to diagnose *Corynebacterium pseudotuberculosis* by enzyme-linked immunoassay

**DOI:** 10.1186/s13568-020-01125-0

**Published:** 2020-10-19

**Authors:** Antonio Pedro Fróes de Farias, José Tadeu Raynal Rocha Filho, Silvana Beutinger Marchioro, Luan Santana Moreira, Andressa Souza Marques, Maria da Conceição Aquino de Sá, Antonio Anderson dos Santos Oliveira, Maria Emília Alcântara, Ricardo Barros Mariutti, Raghuvir Krishnaswamy Arni, Soraya Castro Trindade, Roberto Meyer

**Affiliations:** 1 Immunology and Molecular Biology Laboratory (LABIMUNO), Federal University of Bahia (UFBA), Salvador, BA Brazil; 2 College of Science and Entrepreneurship (FACEMP), Santo Antônio de Jesus, BA Brazil; 3 Feira de Santana State University (UEFS), Feira de Santana, BA Brazil; 4 Multiuser Center for Biomolecular Innovation (IBILCE/UNESP), São José do Rio Preto, SP Brazil

**Keywords:** Caseous lymphadenitis, *Corynebacterium pseudotuberculosis*, ELISA, SodC recombinant protein

## Abstract

Caseous lymphadenitis (CL) is a chronic infectious disease that affects sheep and goats. Many serological tests have been developed to detect the disease; one of the most widely used is the enzyme-linked immunosorbent assay (ELISA), due to its advantages, which include acceptable cost-effectiveness, applicability, sensitivity and specificity. ELISA formulations using recombinant proteins can exhibit significant sensitivity and specificity when using a single purified antigen. DTxR, Trx, TrxR, LexA, SodC, SpaC, NanH, and PknG recombinant proteins can be considered target proteins for ELISA development due to its extracellular or on the cell surface location, which allows a better recognition by the immune system. Therefore, the objectives of this study were to evaluate the antigenic reactivity of *Corynebacterium pseudotuberculosis* recombinant proteins in goat and sheep serum. Of eight proteins evaluated, rSodC was selected for validation assays with small ruminant serum samples from the semiarid region of the state of Bahia, Brazil. Validation assays with goat serum samples showed that ELISA-rSodC presented sensitivity and specificity of 96% and 94%, respectively. Validation assays with sheep serum showed that ELISA-rSodC exhibited sensitivity and specificity of 95% and 98%, respectively. Analysis of 756 field serum samples showed that rSodC identified 95 positive samples (23%) in goats and 75 positive samples (21%) in sheep. The ELISA with recombinant SodC protein developed in this study discriminated positive and negative serum samples with high levels of sensitivity and specificity. This formulation is promising for epidemiological surveys and CL control programs.

*Trial registration* AEC No 4958051018. 12/18/2018, retrospectively registered

## Key points

- rSodC protein was selected for diagnostic tests of sheep and goats.
- ELISA-rSodC showed high levels of sensitivity and specificity.
- Promising for epidemiological research and control of caseous lymphadenitis.

## Introduction

Caseous lymphadenitis (CL) caused by the bacterium *Corynebacterium pseudotuberculosis*, is a chronic infectious disease that affects mainly sheep and goats. As the initial symptoms are not immediately noticeable, numerous serological tests have been developed to detect the infection in asymptomatic animals. The enzyme-linked immunosorbent assay (ELISA) is one of the most widely used due to its advantages of cost-effectiveness, applicability, sensitivity, and acceptable specificity (Menzies et al. 2004; Baird and Fontaine 2007; Oreiby 2015). Currently, there are no reliable tests available to diagnose all CL cases. ELISA, using several antigenic preparations, has already been tested (Sutherland et al. 1987; Menzies et al. 1994; Sting et al. 1998; Carminati et al. 2003); however, few formulations use recombinant proteins (Menzies et al. 1994; Rezende et al. 2016; Barral et al. 2019; Silva et al. 2019). This strategy could significantly increase sensitivity and specificity due to the use of a single purified antigen (Rezende et al. 2016).

The complete sequencing of the *C. pseudotuberculosis* genome, combined with the introduction of more advanced technologies, such as mass spectrometry, brought new perspectives to the study of proteins excreted by microorganisms as potential therapeutic, vaccine, or immunoassay targets (D’Afonseca et al. 2010; Bastos et al. 2012; Araújo et al. 2019). Some of the highlighted immunoassay and vaccine development target proteins include diptheric toxin repressor homologue (DTxR), Trx and TrxR thioredoxin complex proteins, LexA proteins, superoxide dismutase-C (SodC), SpaC, neuraminidase H (NanH), and PknG serine/threonine kinases Trost et al. 2010; Hall et al. 2011; Lin et al. 2016; SantanaJorge et al. 2016).

Diptheric toxin repressor homologue (DTxR) is responsible for regulating iron absorption and inhibiting diphtheria toxin synthesis in several species of the genus *Corynebacterium* (De Zoysa et al. 2005; Oliveira et al. 2017). The proteins of the Trx and TrxR thioredoxin complex detect and respond to oxidative stress generated by cell respiration, metabolism, and immune responses by host cells (Matsuzawa et al. 2017). The LexA protein is a key component of the SOS response, the main regulatory mechanism for DNA repair genes in many bacteria (Smollett et al. 2012).

SodC is an extracellular protein that protects the surface of *C. pseudotuberculosis* cells from the superoxide generated by mammalian host cells (Trost et al. 2010; SantanaJorge et al. 2016), while SpaC protein is recognized as an important virulence factor in bacterial adhesion to host tissues (SantanaJorge et al. 2016). The NanH protein belongs to an extracellular protein class that improves the recognition of sialic acids exposed on animal cell surfaces (Trost et al. 2010; Corrêa et al. 2018), and PknG is a protein possibly involved in glutamine metabolism and inhibition of phagolysosome formation (SantanaJorge et al. 2016).

Considering that the use of an efficient diagnostic method is crucial for the success of infectious disease control programs (Bastos et al. 2012; Rezende et al. 2016), and that extracellular or cell surface antigens allow for better recognition in ELISA tests (Oreiby 2015; Raynal et al. 2018), the objective of this study was to evaluate *C. pseudotuberculosis* rDTxR, rTrx, TrxR, rLexA, rNanH, rSodC, rPknG and rSpaC proteins to develop a CL diagnostic test using serum from naturally and experimentally infected goats and sheep.

## Materials and methods

### Animal serum samples

#### Standardization

Standardization of recombinant proteins (rDTxR, rTrx, rTrxR, rLexA, rNanH, rSodC, rPknG and rSpaC) was performed using 16 goat serum samples, ten positive samples from animals experimentally infected with *C. pseudotuberculosis* as confirmed by isolating bacteria from caseous lesions; and six negative serum samples from animals from CL nonendemic areas. ELISA-rSodC obtained the best results of sensitivity, specificity and differentiation of positive and negative samples in standardization, being selected for the later stages.

#### Validation

In the first validation step, recombinant SodC protein was selected to be tested with positive serum control samples from 50 goats and 45 sheep in which *C. pseudotuberculosis* infection was confirmed by isolating bacteria from caseous lesions. Samples from 50 goats and 45 sheep from CL nonendemic areas in southern Brazil, where there are strict controls for introducing new animals, were used as a negative control (Barral et al. 2019).

The second stage included 756 field samples of small ruminants from the semiarid region of the state of Bahia, Brazil, of which 400 were goat samples and 356 were sheep samples. All samples used had prior confirmation of the presence or absence of humoral response to *C. pseudotuberculosis* by indirect ELISA using bacterium secreted antigens (Carminati et al. 2003).

All procedures involving animals were performed according to the recommendations of the Animal Ethics Committee (AEC) of the Institute of Health Sciences, Federal University of Bahia, under protocol No. 4958051018.

### Antigenic evaluation of recombinant proteins

#### Obtaining recombinant proteins

The nucleotide sequences of the codon-optimized genes were deposited in the NCBI GenBanK BanKit with access numbers: LexA [MT918383]; Trx [MT918384]; TrxR [MT918385]; DTxR [MT918386]; NanH [MT918387]; SodC [MT918388]; PknG [MT918389]; SpaC [MT918390].

The rDTxR, rTrx, rTrxR, and rLexA recombinant proteins used in this study were kindly provided by the Multiuser Center for Biomolecular Innovation, IBILCE/UNESP, São José do Rio Preto, SP, Brazil. These proteins were produced and synthesized as described by Kabsch (2010).

The genes coding for *C. pseudotuberculosis* SodC, SpaC, NanH, and PknG proteins were synthesized and cloned individually in the commercial vector pD444-NH (DNA 2.0 Inc., USA) (https://www.atum.bio), with the original codons replaced by *Escherichia coli*-optimized codons. The recombinant plasmids were transformed into *E. coli* BL21 (DE3) Star and purified using affinity chromatography, as previously described (Simionatto et al. 2010). Fractions containing recombinant proteins were identified using sodium dodecyl sulfate polyacrylamide gel electrophoresis (SDS-PAGE) and quantified using the Lowry protein assay (Bio-Rad Laboratories, CA, USA) and following the manufacturer’s instructions.

#### Indirect ELISA standardization using recombinant proteins

A checkerboard procedure with different antigen concentrations, serum sample dilutions, and anti-IgG goat and sheep antigens tested in combination was used to screen recombinant antigens and standardize ELISA. 96-well polystyrene plates (GREINER Bio-One, São Paulo, Brazil) were sensitized with 100 µL of each of the recombinant proteins (at concentrations of 0.1, 0.5, 1.0, and 2.0 µg/mL), diluted in 0.05 M carbonate/bicarbonate buffer (pH 9.6), and incubated at 4 ºC for 16 h. The plates were washed with 0.01 M PBS, 0.05% Tween 20 (PBS-T) and blocked with 200 µL of casein (5%) per well for two h. After washing with PBS-T, 50 µL/well of control sera (positive and negative) were added at 1:50, 1:100, and 1:200 dilutions in PBS-T containing 1% casein, and incubated at 37 ºC for one h. After being washed five more times, 50 µL/well of the anti-IgG goat or sheep antibody conjugated with peroxidase (Bethyl, Montgomery, USA) was added at dilutions of 1:5,000, 1:10,000, 1:20,000, and 1:30,000 in PBS-T containing 1% casein, and incubated at 37 ºC for 45 min. Color development was performed with the addition of 50 µL/well of 1:2-O-phenylenediamine substrate (OPD) (Sigma-Aldrich, St. Louis, USA) at 22 ºC, away from light, for 20 min. The reaction was arrested by adding 25 µL/well of 4N H_2_SO_4_. The mean optical density (OD) at 492 nm was determined using a microtiter plate reader (THERMO PLACA, Miami, USA).

The interplate OD was corrected for each standardized ELISA mode by multiplying the correction factor (FtC) between plates by the OD reading (Zwirner 1996). The corrected OD (cD) was calculated using the following formula: FtC = mean OD of the standard positive of the reference plate /Mean OD of the standard positive of each plate. Therefore, cD = OD × FtC.

#### ELISA validation with recombinant SodC protein

After identifying the optimal antigen concentration (0.1 µg/mL), serum dilutions (1:100) and goat and sheep secondary antibodies (1:20,000), recombinant SodC protein was selected to validate the ELISA using goat and sheep sera. The ELISA was used with antigens secreted from *C. pseudotuberculosis*, as standardized by Carminati et al. (2003) for comparison. Serum samples were considered positive when the reaction exhibited an OD > mean plus two OD standard deviations obtained for negative controls (Patarroyo et al. 2002).

### Statistical analysis

To evaluate the specificity, sensitivity, and cut-off point of the ELISA immunoassay, the data obtained were analyzed using the Receiver Operating Characteristic (ROC). SPSS software v.23 for Windows was used for statistical analysis. The graphics were generated through the GrapfPad Prism 8 and Microsoft office 2013 package.

## Results

### Recombinant protein expression and purification

**Fig. 1:**
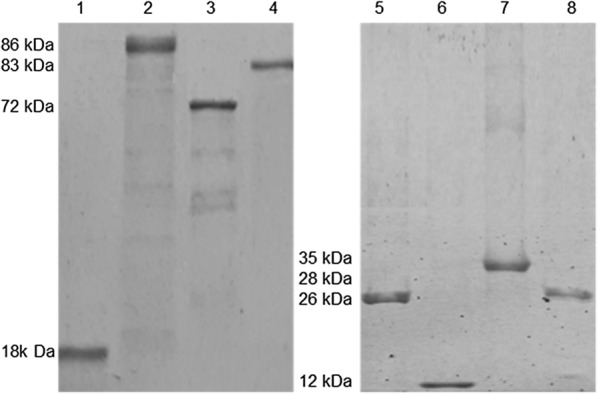
12% SDS-PAGE gel stained with Coomassie blue to show the identification and purification of recombinant proteins. 1-SodC (18 kDa); 2-SpaC (86 kDa); 3-NanH (72 kDa); 4-PknG (83 kDa); 5-DTxR (26 kDa); 6-Trx (12 kDa); 7-TrxR (35 kDa); 8-LexA (28 kDa)

The recombinant proteins expressed in *E. coli* resulted in yields of 1.44 mg/mL (rSodC), 1.0 mg/mL (rPknG), 0.5 mg/mL (rSpaC), and 0.75 mg/mL (rNanH). All recombinant proteins were expressed as insoluble, and were purified under denaturing conditions in 8 M urea. The rDTxR, rTrx, rTrxR, and rLexA recombinant proteins showed yields of 0.55 mg/mL, 1.3 mg/mL, 1.5 mg/mL, and 2.0 mg/mL, respectively. SDS-PAGE (Fig. 1) was used to evaluate the purity of the recombinant proteins used in this study.

### Determination of antigenic potential to screen recombinant proteins

The results obtained for each of the recombinant proteins are shown in Table 1. The highest positive/negative ratio coefficient (9.0) was obtained with rSodC protein at 0.1 µg/mL using serum diluted to 1:100 and secondary antibody at 1:20,000.

**Table 1:** The ratio of OD, serum dilution and conjugated protein values using serum of infected and noninfected goats in indirect checkerboard ELISA using *C. pseudotuberculosis* rDTxR, rTrx, rTrxR, rLexA, rNanH, rSodC, rPknG, and rSpaC recombinant protein as antigens. Pos/neg OD-ratio between positive and negative serum optical density values

Proteins	Pos/neg OD ratio	Serum dilution	Anti-IgG dilution	Concentration (µg/mL^− 1^)
rDTxR	6.5	1:100	1:20.000	0.1
rTrx	6.0			0.1
rTrxR	7.3			0.5
rLexA	5.3			0.1
rNanH	5.4			0.5
rSodC	9.0			0.1
rPknG	3.8			1
rSpaC	1.2			0.5

The results of the ROC curve analysis are presented in Table 2. Only tests performed with rSodC and rPknG proteins showed sensitivity and specificity values > 90%. Although it has high sensitivity (94%) and specificity (97%), rPknG was not chosen for the validation tests due to its low ratio of positive and negative sera (ratio = 3.8) (Table 1). The best sensitivity and specificity values were found for rSodC protein (100% for both), and thus it was selected for validation studies with a greater number of serum samples.

**Table 2:** Cut-off values, sensitivity, and specificity of *C. pseudotuberculosis* rDTxR, rTrx, rTrxR, rLexA, rNanH, rSodC, rPknG, and rSpaC proteins obtained through ROC curve analysis with indirect ELISA results using positive and negative control sera

Proteins	Cut-off	Sensitivity (%)	Specificity (%)
rDTxR	0.272	70	67
rTrx	0.336	90	50
rTrxR	0.224	90	83
rLexA	0.162	70	50
rNanH	0.186	70	67
rSodC	0.275	100	100
rPknG	0.243	94	97
rSpaC	0.162	60	33

### ELISA validation test with recombinant SodC protein

**Fig. 2:**
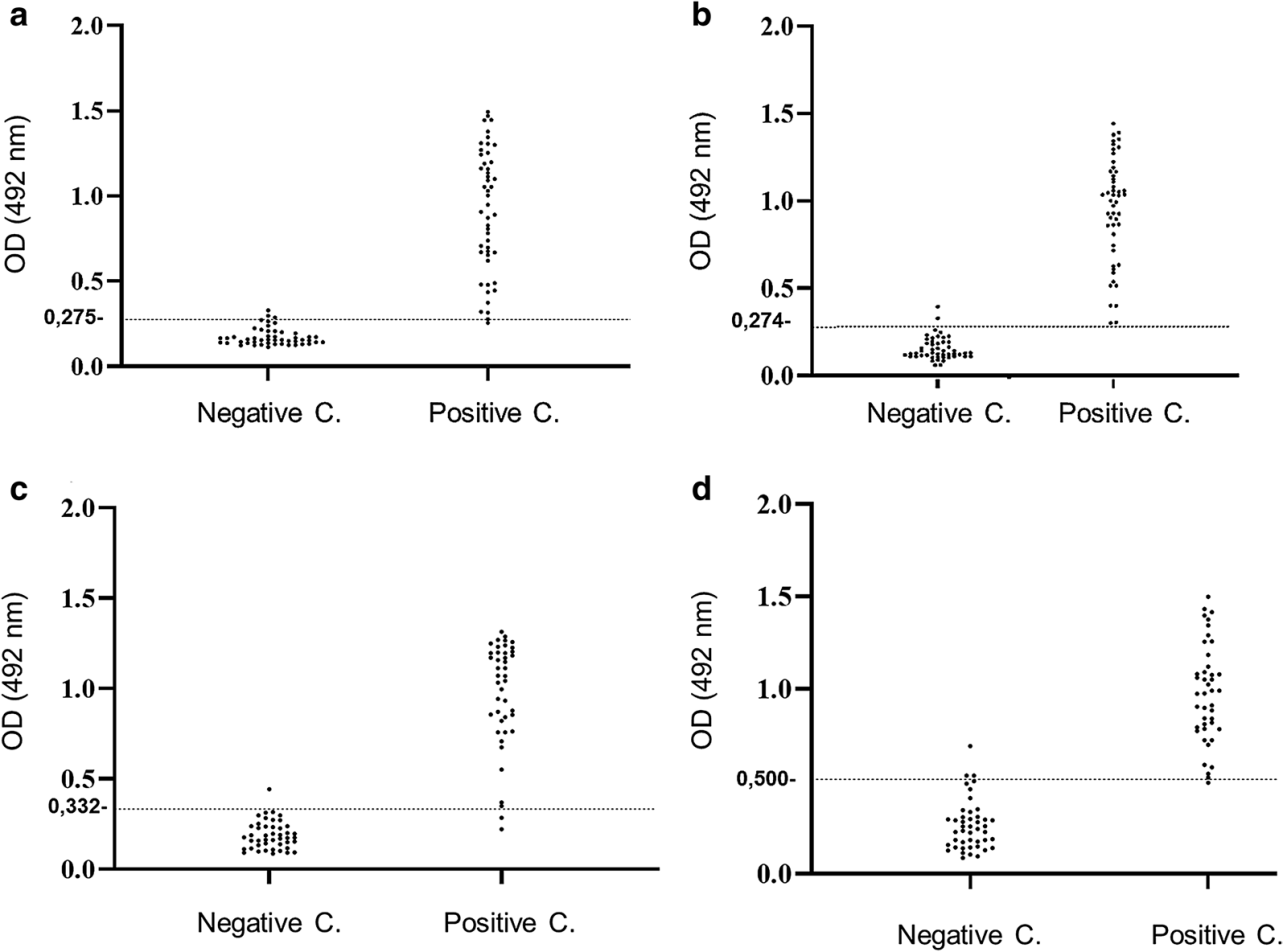
Distribution of OD results from negative and positive control serum samples evaluated by ELISA using rSodC protein antigens and secreted *C. pseudotuberculosis* antigens. **a** ELISA-rSodC with goat sera, **b** ELISA-secreted *C. pseudotuberculosis* antigens with goat sera, **c** ELISA-rSodC with sheep sera and **d** ELISA-secreted *C. pseudotuberculosis* antigens with sheep sera. The graphs show the OD values obtained for each positive or negative sample. The lines in the graphs represent the cut-off value for each test

The rSodC protein was selected for ELISA validation, with a higher number of goat and sheep serum samples previously characterized. Samples from 50 goats and 45 sheep naturally infected were used as positive controls, and samples from 50 goats and 45 sheep from CL nonendemic areas were used as negative controls. The results comparing the ELISA using the recombinant proteins with indirect ELISA using secreted *C. pseudotuberculosis* antigens (Secreted Ag.), are shown in Table 3; Fig. 2.

**Table 3:** Validation parameters for indirect ELISA using positive and negative control serum samples from goats and sheep. rSodC protein and secreted *C. pseudotuberculosis* antigens were used as test antigens

Parameter	Goat		Sheep	
	rSodC	Secreted antigen	rSodC	Secreted antigen
Positive control	50	50	45	45
Negative control	50	50	45	45
Cut-off	0.275	0.274	0.332	0.510
Sensitivity (%)	96	100	95	98
Specificity (%)	94	96	98	93
Accuracy (%)	97.6	99.8	98.9	99.5
Positive predictive value (%)	100	100	100	100
Negative predictive value (%)	93.7	99.5	97.1	98.7

The rSodC protein ELISA differentiated infected from noninfected animals, with sensitivity and specificity levels of 96% and 94%, respectively, for goat samples. These values were similar to those found in testing using secreted *C. pseudotuberculosis* antigens: 100% and 96% sensitivity and specificity, respectively. The standardized rSodC protein ELISA validation with sheep serum exhibited sensitivity and specificity of 95% and 98%, respectively, and in tests using secreted antigens, the sensitivity, and specificity were 98% and 93%, respectively.

In the rSodC protein assay, there were two false-positive results (negative samples with OD above the cut-off point) for goats and one for sheep. As for false-negative results (positive control serum with OD values below the cut-off point), there was one sample for goats and two for sheep. Using the same samples against secreted *C. pseudotuberculosis* antigens, there were two false-positive results for goats, three false-positive results for sheep, and only one false-negative result for sheep.

### Use of the rSodC ELISA in field samples

**Fig. 3:**
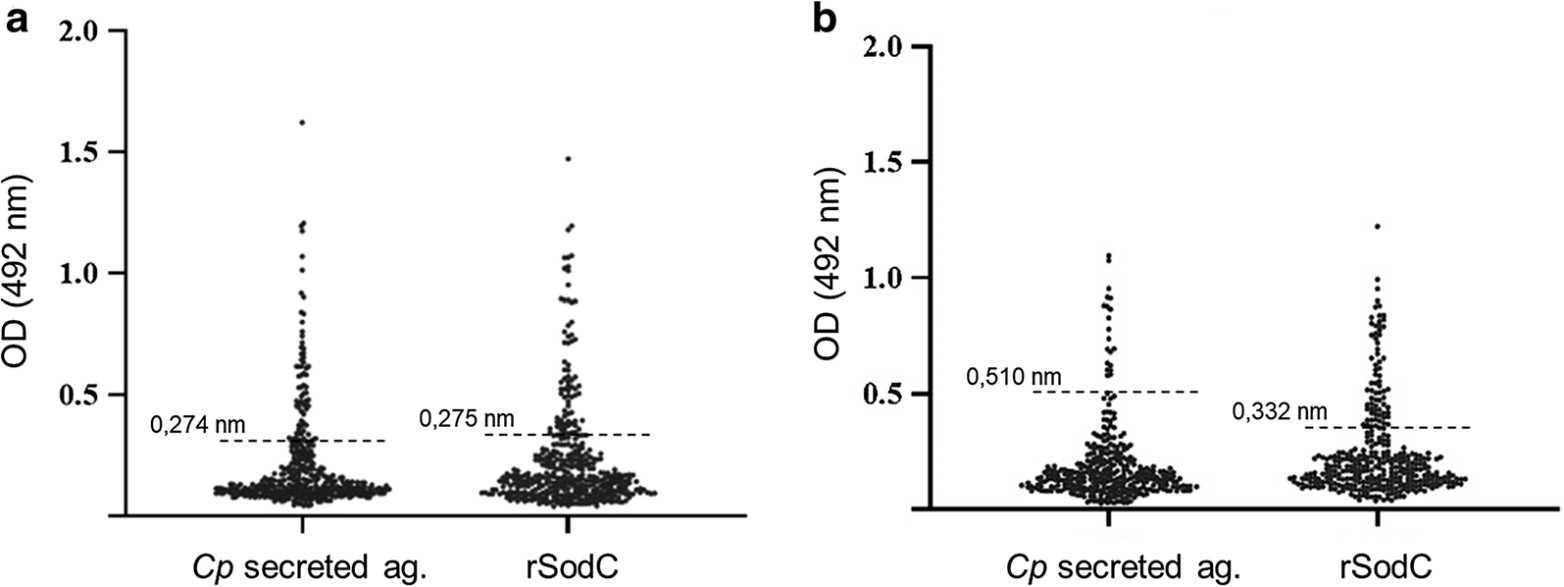
Distribution of OD results using rSodC protein and secreted *C. pseudotuberculosis* antigen from goat (**a**) and sheep (**b**) field samples. The graphs show the OD values obtained for each goat and sheep sample evaluated. The lines in the graphs represent the cut-off value for each test

The ELISA-rSodC was performed using 400 serum sample from goats and 356 from sheep collected in field conditions. The cut-off point was defined by the ROC curve. The results are presented in Fig. 3.

In the ELISA with goat samples, rSodC protein was recognized in the serum of 95 positive samples from the 400 samples evaluated (23%), while the antigen secretion test detected 83 positive samples (20%). For sheep samples, the rSodC protein was recognized in 75 positive samples out of 356 evaluated specimens (21%), while secreted antigens yielded 35 positive results (10%).

## Discussion

In the present study, the standardized test using rSodC protein showed satisfactory performance in the identification of positive and negative cases of *C. pseudotuberculosis* by serological responses, both in the initial standardization tests and in validation tests with goat and sheep samples. The use of this protein as an antigen to diagnose CL is promising due to the observed high sensitivity and specificity values, which were comparable or superior to those described in the literature to detect goats and sheep infected with *C. pseudotuberculosis* using different recombinant antigenic preparations (Menzies et al. 2004; Rezende et al. 2016; Barral et al. 2019).

Sensitivity and specificity are important factors that must be considered when selecting one or more diagnostic tests for a screening program. Serological tests must have sensitivity and specificity > 90% to eliminate CL infections in large batches (O’Reilly et al. 2010). A test of lower specificity may lead to false-positive results, and decreased sensitivity may result in false-negatives (Carminati et al. 2003; Bastos et al. 2012).

The use of rSodC protein presents better ELISA reproducibility conditions when compared to secreted antigens due to greater standardization of antigenic composition. This characteristic is relevant for large-scale testing. As they are purified antigens, recombinant proteins can improve specificity, reducing the chances of cross reactions with molecules of other microorganisms. They can also influence sensitivity, since the amount of immunodominant protein used to sensitize the plaques is greater than the concentration of this molecule in a raw antigen extract (Barral et al. 2019).

For seven (rDTxR, rTrx, rTrxR, rLexA, rNanH, rPknG, and rSpaC) other recombinant proteins of *C. pseudotuberculosis* were evaluated in ELISA, however lower sensitivity and/or specificity were obtained, and were unable to satisfactorily discriminate samples from infected and noninfected animals. These proteins play important roles in the survival of these microorganisms, and have been evaluated *in silico* and *in vitro to* determine the potential of these recombinant constructs as therapeutic targets (Resende et al. 2011; Olson et al. 2013; Lin et al. 2016). However, although they were previously indicated as promising (SantanaJorge et al. 2016), their performance was not confirmed in studies involving animal serum evaluation. The high level of conservation of these proteins in eukaryotes and prokaryotes (Hall et al. 2011) may be the main factor contributing to this performance.

The selection of recombinant antigens for validation tests in the present study was based on standardization with goat serum. The rSodC protein yielded better sensitivity and specificity results. Based on these observations, the conditions used for validation tests in goats were extrapolated for analyses with sheep samples, since the pathogenesis and immune response to the disease caused by *C. pseudotuberculosis* is similar for these animal models.

ELISA is the most widely used serological test to detect *C. pseudotuberculosis* serum status due to its high cost-effectiveness and applicability (Oreiby 2015). Few methods use recombinant proteins to diagnose CL, which can provide significant specificity and sensitivity levels by using a single purified antigen (Rezende et al. 2016). Efforts to characterize the bacterial proteome and to discover new secreted antigens with potential for use in vaccine development and immunoassays against *C. pseudotuberculosis* infection are being made (Raynal et al. 2018). Thus, the good performance of the ELISA test using rSodC protein demonstrated that it is promising for epidemiological research and CL control programs due to its accuracy in detecting goats and sheep infected with *C. pseudotuberculosis.*

## Data Availability

The data of this work are available at: http://www.labimuno.ufba.br/.
